# Network analysis of core symptom changes in hepatocellular carcinoma survivors after transarterial chemoembolization: A longitudinal study

**DOI:** 10.3389/fonc.2026.1960540

**Published:** 2026-10-06

**Authors:** Suping Wang, Xiuyan Wang, Ni Jiang, Yongpei Hu, Lirong Guo, Long Zhang, Yanzhe Liu, Simin Kong, Ying Zhang

**Affiliations:** 1 School of Nursing, Kunming Medical University, Kunming, Yunnan, China; 2 Department of Minimally Invasive Interventional Radiology, The Third Affiliated Hospital of Kunming Medical University (Yunnan Cancer Hospital, Peking University Cancer Hospital Yunnan Hospital), Kunming, Yunnan, China; 3 Department of Gynecology, The Third Affiliated Hospital of Kunming Medical University (Yunnan Cancer Hospital, Peking University Cancer Hospital Yunnan Hospital), Kunming, Yunnan, China; 4 The Third Affiliated Hospital of Kunming Medical University (Yunnan Cancer Hospital, Peking University Cancer Hospital Yunnan Hospital), Kunming, Yunnan, China; 5 Chuxiong Medical College, Chuxiong, Yunnan, China

**Keywords:** core symptoms, health-related quality of life, hepatocellular carcinoma, network analysis, transarterial chemoembolization, unmet supportive care needs

## Abstract

**Objective:**

The aims of this study were to explore the longitudinal trajectories of core symptoms in survivors with hepatocellular carcinoma (HCC) after transarterial chemoembolization (TACE) and to analyze their associations with health-related quality of life (HRQoL) and unmet supportive care needs.

**Methods:**

This longitudinal study enrolled 271 HCC survivors undergoing TACE in China. Patient-reported outcomes were assessed at three time points—24 h (T1), 7 days (T2), and 1 month (T3) post-TACE—using four validated instruments: the MD Anderson Symptom Inventory–Chinese Version (MDASI-C), the Tumor Symptom Measure for Primary Liver Cancer (TSM-PLC), the Quality of Life Instrument for Liver Cancer (QOL-LC V2.0), and the Short Form of Survivors’ Unmet Needs Scale (SF-SUNS). Exploratory factor analysis was conducted to extract discrete symptom clusters, and network analysis was used to calculate strength centrality (rs)—an index quantifying the sum of absolute edge weights connecting each symptom to all others—to identify core symptoms.

**Results:**

Five symptom clusters were identified at T1: Fatigue-Cognitive, Post-embolization, Somatic, Emotional, and Hepatobiliary-Specific. The Post-embolization cluster was present only at T1, whereas the remaining four clusters persisted at T2 and T3. However, the core symptom shifted over time: nausea at T1 (rs = 1.154), lack of appetite at T2 (rs = 1.229), and sadness at T3 (rs = 1.107). HRQoL was lowest at T1, improved at T2, and declined at T3, with all pairwise comparisons significant (all *p* < 0.001). Unmet needs were highest at T3, with all time-point differences significant (all *p* < 0.001). The largest gaps were observed in information provision, care continuity, and emotional support. Greater symptom severity was significantly associated with poorer HRQoL at all time points (all *p* < 0.01).

**Conclusions:**

During the first month post-TACE, the sequence of core symptoms transitioned from nausea to lack of appetite and then to sadness, suggesting time-specific supportive care strategies—including antiemetic prophylaxis, nutritional support, and psychosocial care. Routine assessment of unmet needs may help reduce symptom burden and optimize long-term HRQoL for these survivors.

## 1 Introduction

Hepatocellular carcinoma (HCC) is a leading cause of cancer-related mortality worldwide. In 2022, approximately 865,000 new cases and 758,000 deaths were recorded globally, ranking HCC sixth in incidence and third in cancer-related mortality. China bears a disproportionate burden, accounting for 42.4% of global cases and 41.8% of deaths (1).

Because early-stage HCC is often asymptomatic, most HCC survivors are diagnosed at advanced stages. For HCC survivors with unresectable intermediate-stage disease, transarterial chemoembolization (TACE) is the standard first-line therapy (2). TACE causes local hepatic inflammatory injury and postoperative adverse reactions, triggering a cascade of physical and psychological distress throughout the recovery period. After TACE, HCC survivors frequently experience a range of physical symptoms—including pain, fever, jaundice, and nausea—alongside psychological symptoms such as anxiety and sadness (3). These symptoms tend to co-occur and interact, forming distinct symptom clusters, defined as stable groups of two or more interrelated and concurrent symptoms (4). The synergistic effects of symptom clusters can substantially amplify their negative impact, not only increasing the economic burden of disease but also potentially compromising treatment adherence and long-term survival, and significantly impairing health-related quality of life (HRQoL). Evidence suggests that cancer survivors with more numerous and severe symptoms report worse quality of life and physical functioning (5).

However, most existing studies have focused on individual symptoms and their management, overlooking the interconnections and dynamic evolution among symptoms. Moreover, longitudinal evidence on symptom trajectories in HCC survivors after TACE remains insufficient (6). The majority of prior investigations adopt a cross-sectional design, which can only capture static symptom characteristics at a single time point, failing to track structural changes in symptom networks from the acute postoperative phase to early survivorship (7–9). Unmet supportive care needs have been increasingly recognized as a critical factor influencing HRQoL in cancer survivors, with previous studies documenting substantial unmet needs across physical, psychological, informational, and healthcare service domains (10). Studies have found that 59.4% of patients with HCC reported unmet psychological care needs shortly after admission (11). Such unmet needs are frequently accompanied by financial toxicity and elevated caregiver-related hardship, further compounding the disease-related burden for patients with HCC (12). However, the extent to which such needs emerge and evolve in relation to symptom burden and HRQoL during the post-TACE recovery period has not been adequately examined.

Symptom network analysis offers a novel framework to address these gaps. Grounded in the network theory of psychopathology, this approach conceptualizes symptoms as interconnected nodes, visualizes inter-symptom relationships, tracks temporal shifts, and identifies core and bridge symptoms through centrality metrics (13).

This longitudinal study therefore aimed to (1) identify symptom clusters and core symptoms in HCC survivors post-TACE using network analysis (2); explore the dynamic associations between symptom clusters, HRQoL, and unmet needs across three post-TACE time points [24 h (T1), 7 days (T2), and 1 month (T3)], to inform stage-specific symptom management strategies.

## 2 Materials and methods

### 2.1 Study design and participants

This prospective longitudinal study was conducted at the Third Affiliated Hospital of Kunming Medical University (Yunnan Cancer Hospital) from December 2025 to June 2026. Patients with histopathologically confirmed primary HCC who had undergone TACE were recruited through convenience sampling. Inclusion criteria were as follows (1): histopathologically confirmed primary HCC who had undergone TACE treatment (2), aged ≥18 years and able to give written informed consent, and (3) maintained clear consciousness without dementia or psychiatric disorders. Exclusion criteria were as follows (1): documented history of psychiatric disorders or verbal communication impairments, and (2) severe dysfunction of major organs (heart, brain, and kidneys). The sample size was calculated based on established principles for constructing network models (14). All follow-up assessments (T1–T3) were completed within the first month after the index TACE procedure, prior to any scheduled second-TACE retreatment; no patients received repeated TACE during the observation window. In addition, participants were not restricted from receiving concurrent anti-tumor regimens, including targeted therapy or immunotherapy during the study period.

For a network containing P nodes, the minimum required sample size follows the formula P(P − 1)/2 to ensure adequate statistical power. With 19 symptom nodes included in this study, the calculated minimum sample size was 171. Accounting for a 20% attrition rate, the target sample size was 214 cases. The actual enrolled sample of 271 cases exceeded this requirement, satisfying the statistical demands for network analysis. In addition, the N/p ratio of 14.3 (271/19) also exceeded the widely recommended heuristic threshold of 10 (15), supporting sufficient power for stable and reliable edge-weight and centrality estimation.

### 2.2 Data collection

Assessments were conducted at three post-TACE time points: 24 h (T1), 7 days (T2), and 1 month (T3). Baseline demographic and clinical data were collected upon hospital admission. The study protocol was approved by the Medical Ethics Committee of Kunming Medical University (Approval No. KMMU2025MEC369). After receiving a full explanation of the study, all participants provided written informed consent.

### 2.3 Instruments

#### 2.3.1 General information questionnaire

We collected two categories of baseline data: sociodemographic characteristics (sex, age, educational attainment, marital status, and monthly household per-capita income) and disease-related clinical information (HCC duration, TNM stage, viral hepatitis history, liver cirrhosis history, Child–Pugh classification, and number of prior TACE sessions).

#### 2.3.2 MD Anderson symptom inventory–Chinese version

Symptom severity was assessed using the MD Anderson Symptom Inventory–Chinese Version (MDASI-C), a validated 13-item scale covering common cancer symptoms (16). Each item is rated on an 11-point Likert scale from 0 (no symptoms) to 10 (worst imaginable), with higher scores indicating greater symptom burden. To capture HCC-specific symptoms, we additionally administered the Tumor Symptom Measure for Primary Liver Cancer (TSM-PLC), which includes six items (jaundice, itching, feeling bloated, diarrhea, weight loss, and fever) (17). The scoring of the TSM-PLC is identical to that of the MDASI-C (0–10 scale). The MDASI-C showed acceptable reliability (Cronbach’s α: 0.82–0.94), and the TSM-PLC had a Cronbach’s α of 0.835.

#### 2.3.3 Quality of life instrument for liver cancer

HRQoL was assessed using the Quality of Life Instrument for Liver Cancer (QOL-LC V2.0), developed by Wan et al. (18). It covers four domains: physical functioning (six items), psychological functioning (six items), disease symptoms and treatment side effects (five items), and social functioning (five items). Items are scored from 0 (extreme distress) to 10 (no distress), with higher total scores indicating better HRQoL. Cronbach’s α for this scale ranged from 0.68 to 0.81.

#### 2.3.4 Chinese version of the short form of survivors’ unmet needs scale

Unmet supportive care needs were measured using the Chinese version of the Short Form of Survivors’ Unmet Needs Scale (SF-SUNS), validated by Yan et al. (19). This scale covers four domains: information (3 items), work and finances (8 items), transitional care (6 items), and emotional and psychological support (13 items). Responses are recorded on a five-point Likert scale from 0 to 4, with higher scores indicating greater unmet needs. Cronbach’s α for this scale was 0.89.

### 2.4 Statistical methods

Descriptive data were analyzed using IBM SPSS Statistics for Windows, Version 25.0 (IBM Corp., Armonk, NY, USA). Continuous variables are presented as mean ± standard deviation (SD), and categorical variables are presented as frequencies and percentages.

Exploratory factor analysis (EFA) with varimax rotation was performed on symptoms with an incidence rate ≥20% across the three time points. Data adequacy for EFA was judged via the Kaiser–Meyer–Olkin (KMO) index and Bartlett’s test of sphericity; analyses were considered appropriate when KMO > 0.6 and the sphericity test yielded a statistically significant result. Factors with eigenvalues ≥1.0 and factor loadings >0.4 were retained for symptom clusters.

EFA and network analysis serve complementary roles. EFA identifies latent symptom clusters based on shared variance among symptoms, providing a dimensional framework of symptom co-occurrence. Network analysis then examines the interconnections among individual symptoms at the observational level, identifying core symptoms (high strength centrality) and bridge symptoms (high bridge strength) that may drive symptom propagation within and between clusters. In the present analysis, these EFA-derived symptom clusters were adopted as the cluster membership input to calculate bridge-centrality indices.

Symptom networks at T1, T2, and T3 were constructed using the EBICglasso function via the qgraph package in R, based on Spearman correlation matrices. In the symptom network, nodes represent symptoms, and edges represent associations between two symptoms; thicker edges indicate stronger correlations. Centrality indices—including strength centrality, closeness centrality, and expected influence—were calculated for each node. Bridge centrality indices were calculated using the networktools package to identify bridge symptoms, with higher bridge strength values indicating that a symptom serves as a key node connecting different symptom clusters. Node predictability was assessed using the mgm package, reflected by the coefficient of determination (*R*²) for each node. Network accuracy was evaluated using the bootnet package, which calculated 95% confidence intervals for edge weights based on bootstrap resampling. Network stability was assessed via the case-dropping subset bootstrap method (1,000 resamples), with a correlation stability (CS) coefficient >0.25 considered acceptable and >0.50 indicating good stability (20). Because the CS coefficients for closeness centrality and betweenness centrality fell below the acceptable threshold, only the CS coefficient for strength centrality was used to evaluate network stability (21). No formal inferential statistical comparisons of overall network structure were conducted across the three time points (T1, T2, and T3). Observed differences in network patterns and core-symptom locations over time were interpreted descriptively based on visual inspection of network plots.

Of the 299 patients enrolled at baseline, 271 (90.6%) completed all three assessments and were included in the final analysis. Missing data were handled using complete-case analysis, as the proportion of missing data was less than 10% and the missingness was considered random.

Repeated-measures analysis of variance (RM-ANOVA) was used to compare HRQoL across the three follow-up time points; a separate RM-ANOVA was performed for unmet supportive care needs. Before performing RM-ANOVA, the sphericity assumption was tested using Mauchly’s test. When the sphericity assumption was violated (Mauchly’s test *p* < 0.05), the Greenhouse–Geisser correction was applied to adjust the degrees of freedom. *Post-hoc* pairwise comparisons were adjusted using Bonferroni correction. Spearman’s rank correlations were used to test associations between symptom clusters and HRQoL at each time point.

## 3 Results

### 3.1 General and clinical characteristics of HCC survivors

Of the total 299 patients who completed baseline evaluation at T1 (24 h post-TACE), 100% of participants submitted valid questionnaires. At T2 (7 days post-TACE), valid responses were obtained from 283 patients, corresponding to a response rate of 94.65%; 16 participants were lost to follow-up during this interval, including 4 deaths, 2 voluntary withdrawals, and 10 unreachable patients. At T3 (1 month post-TACE), valid questionnaires were collected from 271 patients, with a response rate of 95.76% relative to the T2 sample; an additional 12 participants withdrew before T3, comprising 5 deaths, 3 individuals who refused repeated surveys, and 4 patients lost to contact. Ultimately, 271 participants finished all three waves of assessments. All detailed dropout records, including specific loss reasons and corresponding case numbers, were fully documented in our clinical follow-up files.

Among the 271 HCC survivors with complete three-wave data, 202 (74.5%) were male and 69 (25.5%) were female, with a mean age of 56.77 ± 10.22 years. Regarding TNM stage, 145 (53.5%) were stage III. The majority of participants had Child–Pugh class A liver function (242, 89.3%). A history of viral hepatitis was reported in 159 HCC survivors (58.7%), and 88 (32.5%) had concurrent liver cirrhosis. For prior TACE sessions, 78 HCC survivors (28.8%) had received one to two sessions, 113 (41.6%) had three to five sessions, and 80 (29.5%) had more than five sessions. Detailed sociodemographic and clinical data are presented in **Table 1**.

**Table 1:** Demographic and clinical characteristics of HCC survivors (*N* = 271).

Variables	*n* (%)
Sex	
Male	202 (74.5%)
Female	69 (25.5%)
Age (years)	
<45	29 (10.7%)
45–59	141 (52%)
60–74	91 (33.6%)
75–89	10 (3.7%)
Education level	
Primary school and below	101 (37.3%)
Junior high school	94 (34.7%)
High school	43 (15.9%)
University degree or above	33 (12.2%)
Marital status	
Married	249 (91.9%)
Single	6 (2.2%)
Divorced or widowed	16 (5.9%)
Monthly per-capita household income (yuan)	
<3,000	155 (57.2%)
3,000–5,000	92 (33.9%)
>5,000	24 (8.9%)
Tumor staging	
I	19 (7%)
II	68 (25.1%)
III	145 (53.5%)
IV	39 (14.4%)
History of hepatitis viruses	
Yes	159 (58.7%)
No	112 (41.3%)
Cirrhosis of the liver	
Yes	88 (32.5%)
No	183 (67.5%)
Duration of cancer survivorship (months)	
<1	61 (22.5%)
1–6	67 (24.7%)
6–12	37 (13.7%)
>12	106 (39.1%)
Child–Pugh grade	
A	242 (89.3%)
B	29 (10.7%)
Comorbidity history	
Yes	58 (21.4%)
No	213 (78.6%)
Number of previous TACE treatments	
0–2	108 (39.9%)
v3–5	113 (41.6%)
v>5	50 (18.5%)

To evaluate potential attrition bias, baseline characteristics were compared between completers (*n* = 271) and non-completers (*n* = 28). The two groups were similar in most demographic and clinical variables (all *p* > 0.05), except for age (*p* = 0.017). The overall attrition rate was 9.4%, and the majority of baseline characteristics were balanced, suggesting minimal attrition bias.

### 3.2 Symptom characteristics of HCC survivors

The incidence and severity of symptoms at each time point are presented in **Table 2**. At T1, the most prevalent symptoms were fatigue (92.3%) and disturbed sleep (90.4%), with disturbed sleep showing the highest severity score (5.38 ± 2.71). At T2, fatigue remained the most prevalent symptom (87.1%), followed by disturbed sleep (81.2%); lack of appetite (4.21 ± 2.75) and fatigue (4.01 ± 2.51) had the highest severity scores. At T3, distress became the most prevalent symptom (86.0%), and sadness was also among the most common (75.6%); fatigue was the most severe symptom (3.90 ± 2.48).

**Table 2:** Symptom incidence and severity among HCC survivors (*N* = 271).

Symptom	T1-*N* (%)	T1-Mean ± Standard Deviation	T2-*N* (%)	T2-Mean ± Standard Deviation	T3-*N* (%)	T3-Mean ± Standard Deviation
Pain	207 (76.4)	3.97 ± 3.12	145 (53.5)	3.97 ± 3.12	96 (35.4)	1.23 ± 1.90
Fatigue	250 (92.3)	4.03 ± 2.48	236 (87.1)	4.01 ± 2.51	232 (85.6)	3.90 ± 2.48
Nausea	193 (71.2)	3.42 ± 3.04	118 (43.5)	3.31 ± 3.12	79 (29.2)	0.90 ± 1.66
Disturbed sleep	245 (90.4)	5.38 ± 2.71	220 (81.2)	3.33 ± 2.32	204 (75.3)	3.23 ± 2.67
Distress	227 (83.8)	3.04 ± 2.10	208 (76.8)	3.04 ± 2.10	233 (86.0)	3.06 ± 1.95
Shortness of breath	86 (31.7)	1.03 ± 1.85	109 (40.2)	1.03 ± 1.85	89 (32.8)	0.97 ± 1.63
Difficulty remembering	117 (43.2)	1.46 ± 2.13	84 (31.0)	1.46 ± 2.13	98 (36.2)	1.18 ± 1.89
Lack of appetite	210 (77.5)	3.94 ± 3.01	213 (78.6)	4.21 ± 2.75	179 (66.1)	2.79 ± 2.69
Drowsiness	171 (63.1)	1.96 ± 2.03	119 (43.9)	1.96 ± 2.03	120 (44.3)	1.38 ± 1.85
Dry mouth	153 (56.5)	2.62 ± 2.72	59 (21.8)	2.62 ± 2.73	41 (15.1)	0.58 ± 1.51
Sadness	175 (64.6)	2.09 ± 2.10	160 (59.0)	2.09 ± 2.11	205 (75.6)	2.45 ± 2.00
Vomiting	114 (42.1)	2.24 ± 3.20	48 (17.7)	2.24 ± 3.20	33 (12.2)	0.32 ± 1.02
Numbness	86 (31.7)	1.13 ± 1.98	50 (18.5)	1.13 ± 1.98	52 (19.2)	0.56 ± 1.31
Feeling bloated	110 (40.6)	1.69 ± 2.55	122 (45.0)	1.69 ± 2.55	120 (44.3)	1.43 ± 2.02
Diarrhea	50 (18.5)	0.63 ± 1.61	65 (24.0)	0.62 ± 1.61	102 (37.6)	1.24 ± 1.89
Weight loss	229 (84.5)	3.83 ± 2.87	22 (8.1)	3.83 ± 2.87	118 (43.5)	1.17 ± 1.67
Jaundice	148 (54.6)	1.99 ± 2.36	159 (58.7)	1.98 ± 2.20	147 (54.2)	1.88 ± 2.25
Itching	125 (46.1)	1.44 ± 2.01	141 (52.0)	1.44 ± 2.01	153 (56.5)	1.85 ± 2.07
Fever	117 (43.2)	1.41 ± 1.99	35 (12.9)	1.41 ± 1.99	16 (5.9)	0.24 ± 1.05

### 3.3 Extraction of symptom clusters in HCC survivors

We performed EFA on symptoms with a prevalence of ≥20% at any time point, retaining items with factor loadings ≥0.4 (22).

At T1, five symptom clusters were extracted: the Post-embolization Symptom Cluster (pain, nausea, vomiting, and fever), the Somatic Symptom Cluster (disturbed sleep, lack of appetite, drowsiness, feeling bloated, and weight loss), the Fatigue-Cognitive Symptom Cluster (fatigue, shortness of breath, difficulty remembering, dry mouth, and numbness), the Emotional Symptom Cluster (distress and sadness), and the Hepatobiliary-Specific Symptom Cluster (jaundice and itching). The cumulative variance explained was 60.30%.

At T2, four symptom clusters were extracted: the Somatic Symptom Cluster (disturbed sleep, lack of appetite, drowsiness, feeling bloated, pain, nausea, and diarrhea), the Fatigue-Cognitive Symptom Cluster (fatigue, shortness of breath, difficulty remembering, and dry mouth), the Emotional Symptom Cluster (distress and sadness), and the Hepatobiliary-Specific Symptom Cluster (jaundice and itching). The cumulative variance explained was 61.43%.

At T3, four symptom clusters were extracted: the Somatic Symptom Cluster (disturbed sleep, lack of appetite, drowsiness, feeling bloated, weight loss, pain, nausea, and diarrhea), the Fatigue-Cognitive Symptom Cluster (fatigue, shortness of breath, and difficulty remembering), the Emotional Symptom Cluster (distress and sadness), and the Hepatobiliary-Specific Symptom Cluster (jaundice and itching). The cumulative variance explained was 61.79%. Factor loadings and cluster assignments for each symptom at each time point are presented in **Table 3**.

**Table 3:** Factor loadings of symptom items in HCC survivors with hepatocellular carcinoma (*N* = 271).

Symptom Cluster	T1-Symptom	T1-Factor Loading	T1-Symptom	T1-Factor Loading	T1-Symptom	T1-Factor Loading
Somatic symptom cluster	Disturbed sleep	0.709	Disturbed sleep	0.647	Disturbed sleep	0.588
	Lack of appetite	0.578	Lack of appetite	0.767	Lack of appetite	0.701
	Drowsiness	0.496	Drowsiness	0.620	Drowsiness	0.593
	Feeling bloated	0.638	Feeling bloated	0.626	Feeling bloated	0.616
	Weight loss	0.702	—		Weight loss	0.500
	—		Pain	0.473	Pain	0.597
	—		Nausea	0.655	Nausea	0.704
	—		Diarrhea	0.565	Diarrhea	0.532
Fatigue-cognitive symptom cluster	Fatigue	0.689	Fatigue	0.584	Fatigue	0.452
	Shortness of breath	0.709	Shortness of breath	0.716	Shortness of breath	0.521
	Difficulty remembering	0.652	Difficulty remembering	0.850	Difficulty remembering	0.860
	Dry mouth	0.478	Dry mouth	0.517	—	
	Numbness	0.511	—		—	
Emotional symptom cluster	Distress	0.888	Distress	0.895	Distress	0.888
	Sadness	0.881	Sadness	0.891	Sadness	0.861
Hepatobiliary-specific symptom cluster	Jaundice	0.878	Jaundice	0.899	Jaundice	0.902
	Itching	0.833	Itching	0.907	Itching	0.901
Post-embolization symptom cluster	Pain	0.599	—		—	
	Nausea	0.901	—		—	
	Vomiting	0.873	—		—	
	Fever	0.607	—		—	

### 3.4 Symptom networks and core symptoms over time

Symptom networks at the three post-TACE time points are visualized in **Figure 1**. All network models were constructed using regularized partial correlation coefficients. Bootstrap validation yielded narrow confidence intervals for all edge weights, indicating good network stability at each time point (**Figure 2**). CS coefficients were calculated to evaluate network robustness. CS values for strength centrality and expected influence (EI) at T1, T2, and T3 were 0.596, 0.515, and 0.519, respectively, indicating ideal stability (CS > 0.50). Stability distributions are displayed in **Figure 3**.

**Figure 1:**
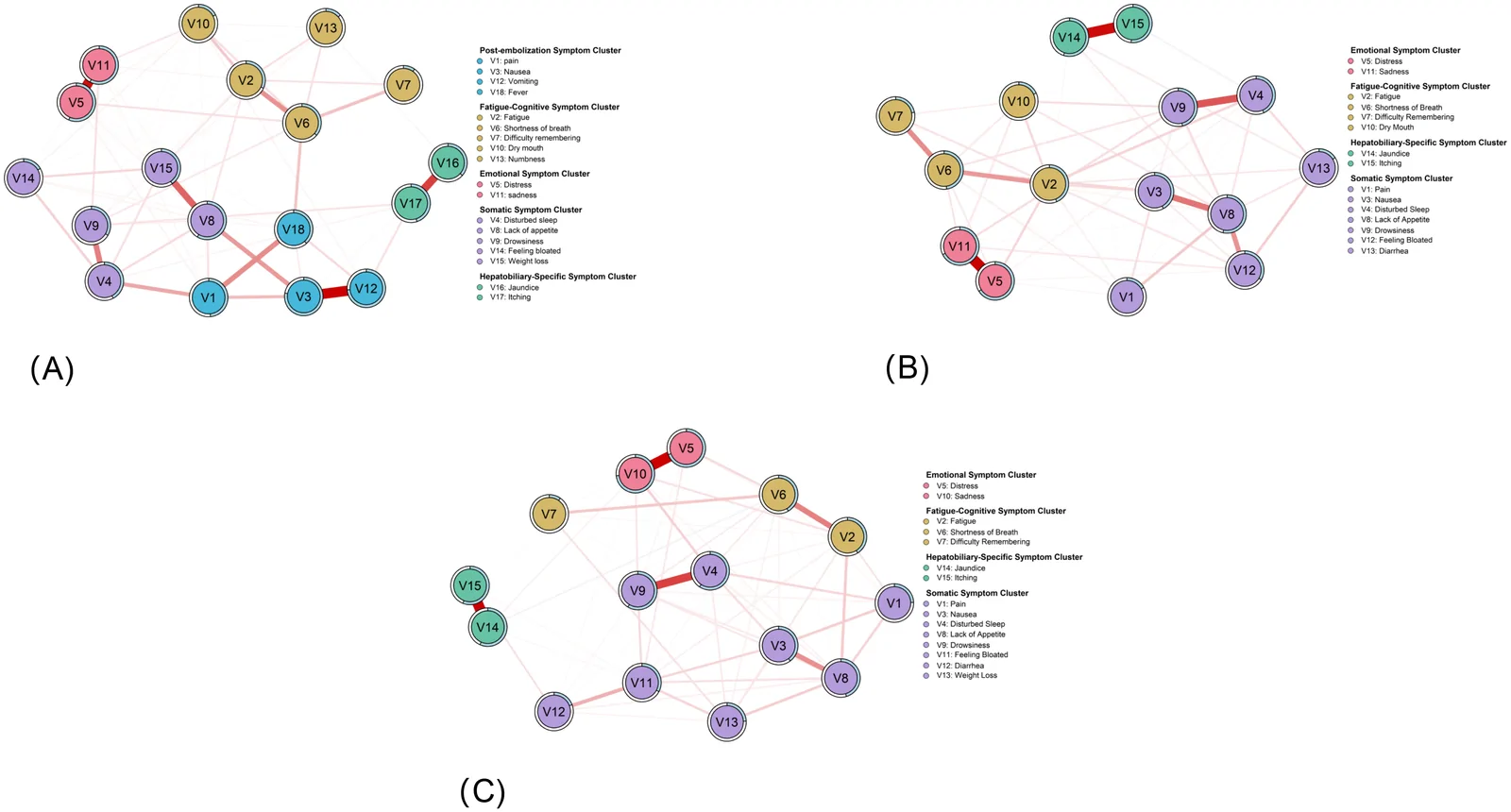
Symptom network of HCC survivors at T1 (24 h post-TACE), T2 (7 days post-TACE), and T3 (1 month post-TACE). **(A)** T1; **(B)** T2; **(C)** T3.

**Figure 2:**
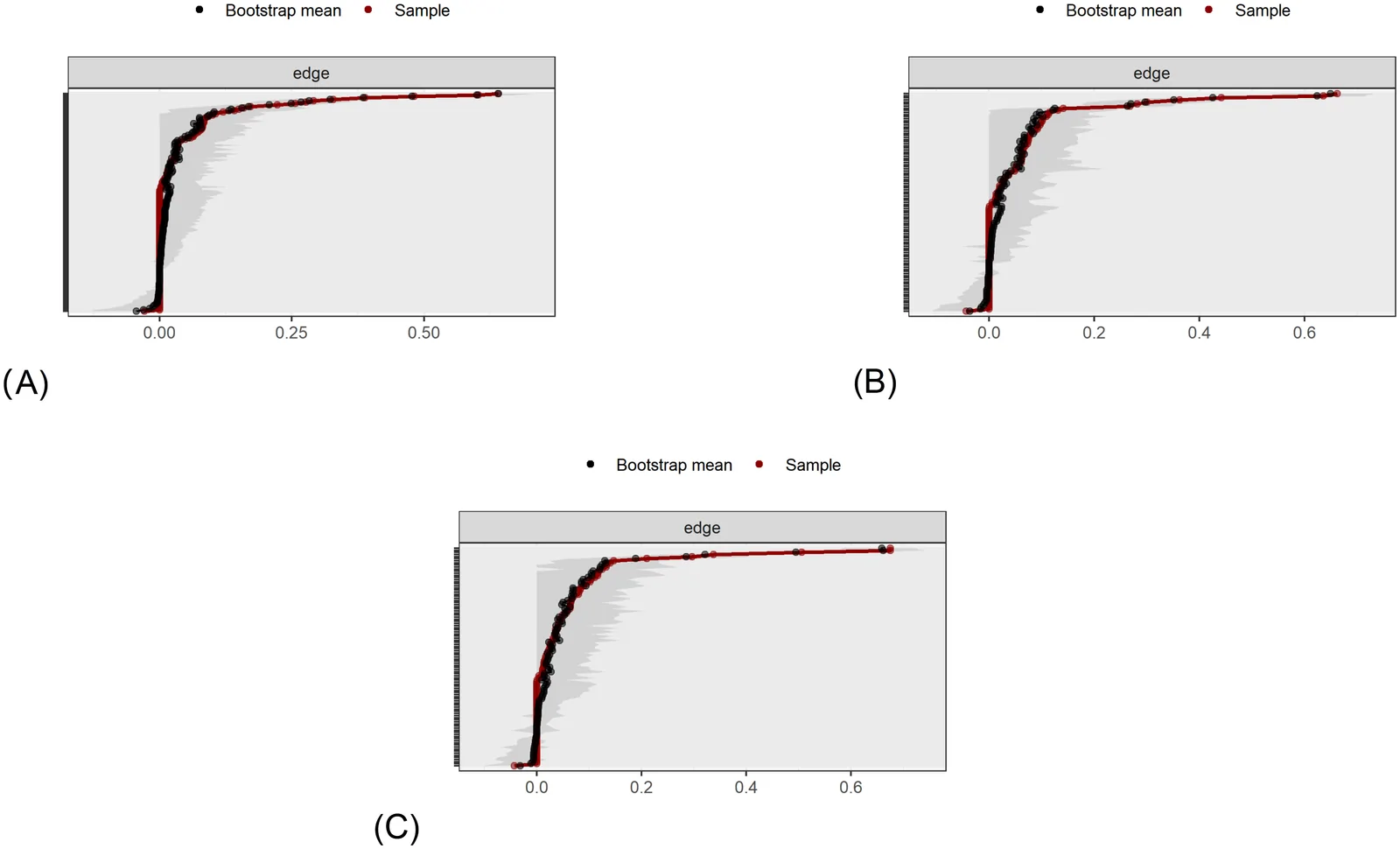
Bootstrap analysis of edge weight accuracy for the symptom networks at T1 **(A)**, T2 **(B)**, and T3 **(C)**. Gray bands denote 95% confidence intervals; narrower bands indicate higher precision.

**Figure 3:**
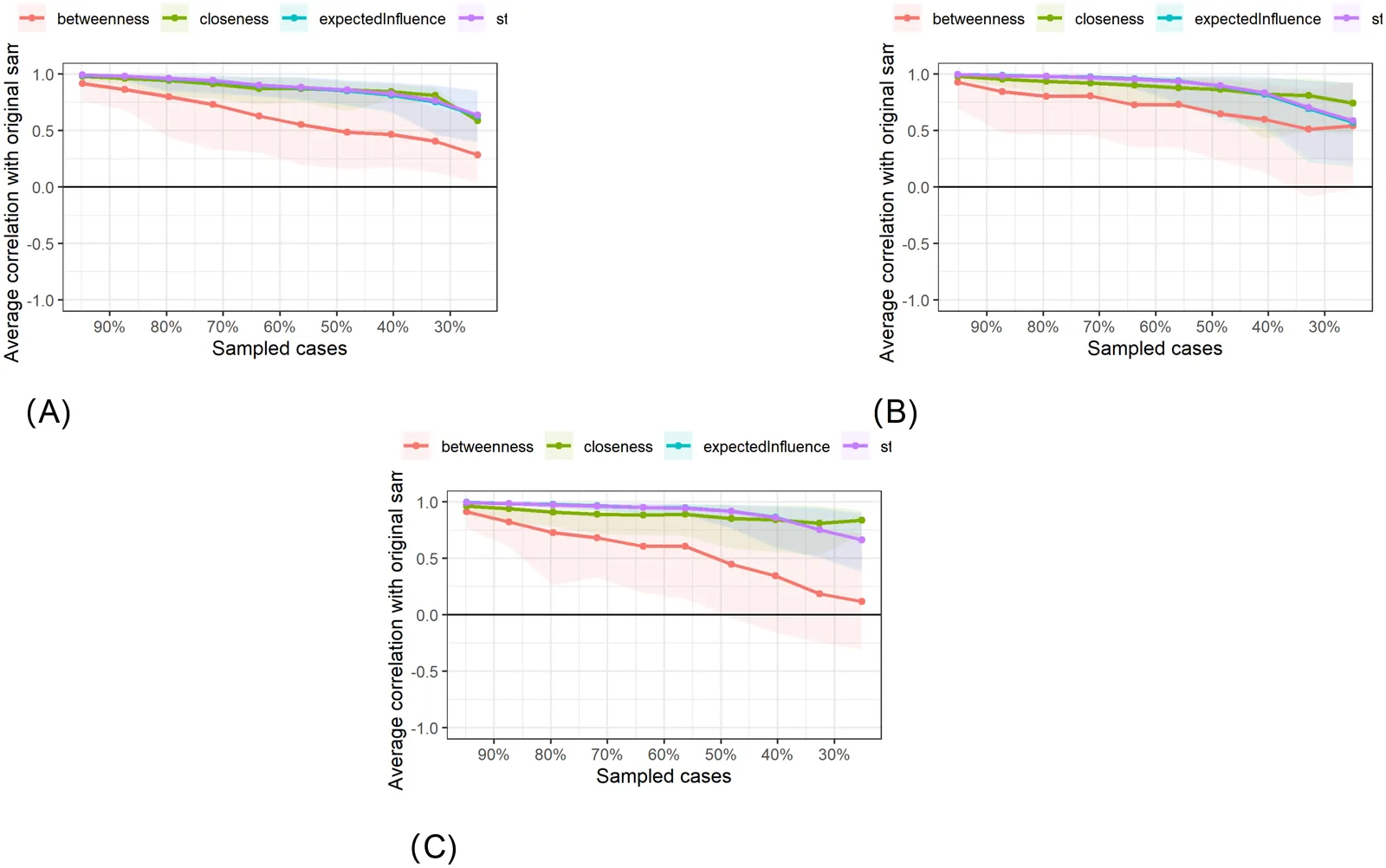
Stability analysis of centrality indices for the symptom networks at T1 **(A)**, T2 **(B)**, and T3 **(C)**.

Positive correlations predominated among pairwise symptom associations. The strongest symptom pairs varied over time: nausea–vomiting at T1 (*r* = 0.640), distress–sadness at T2 (*r* = 0.662), and distress–sadness at T3 (*r* = 0.675). Core symptoms shifted substantially across the recovery phases. At T1, nausea was the network core with the highest strength centrality (rs = 1.154) and high node predictability (*R*² = 0.779); lack of appetite was the primary bridge node (rbs = 3.767). At T2, lack of appetite replaced nausea as the central symptom (rs = 1.229), and sadness emerged as the primary bridge node (rbs = 3.499). At T3, sadness reached its peak strength centrality (rs = 1.107) and exerted the strongest bridging effect (rbs = 4.140), acting as the central hub of the symptom network. Strength centrality distributions are shown in **Figure 4**.

**Figure 4:**
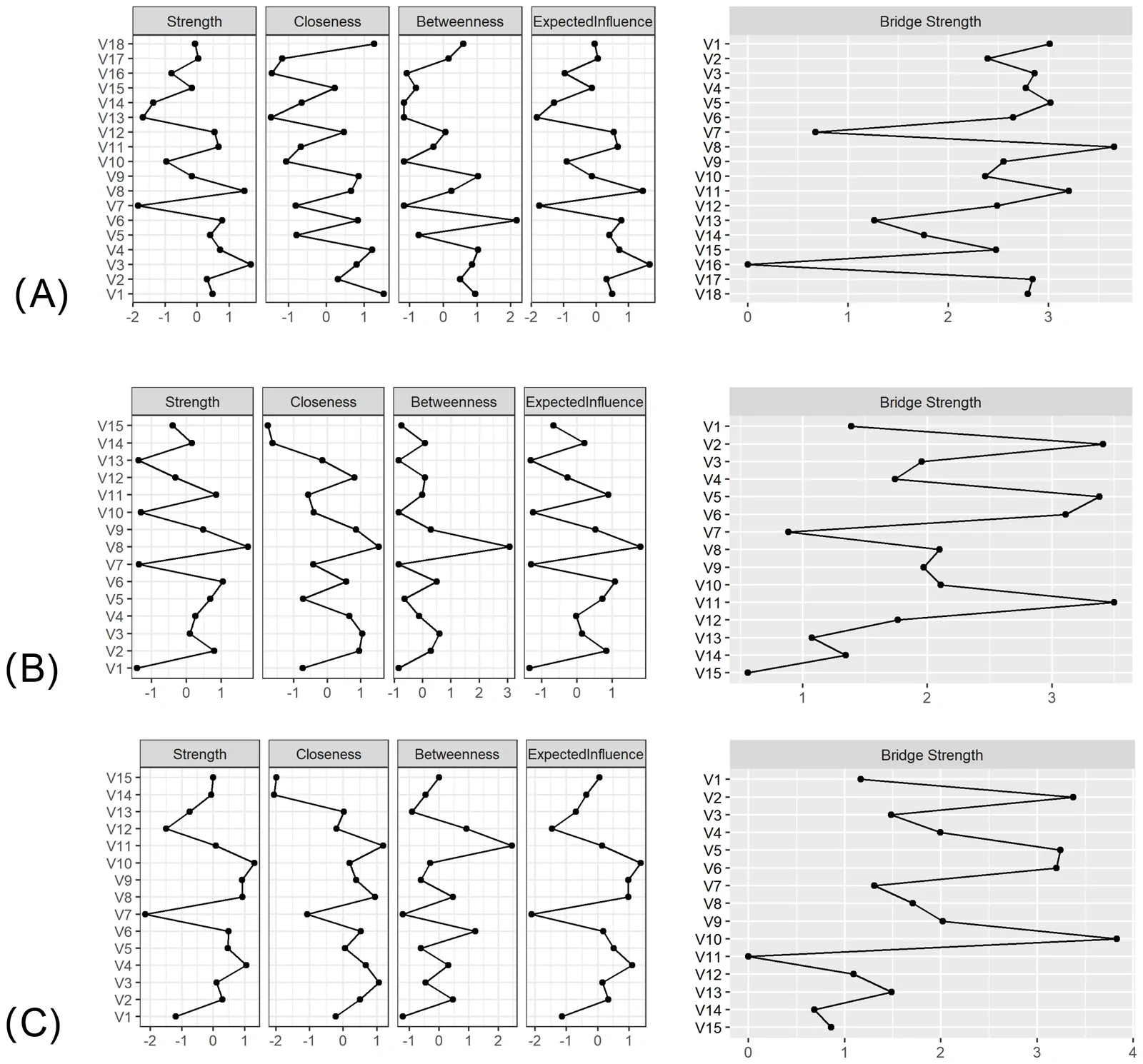
Centrality indices of the symptom network at T1 **(A)**, T2 **(B)**, and T3 **(C)**. Strength centrality (rs) is shown on the left and bridge strength (rbs) on the right.

### 3.5 Longitudinal variations in health-related quality of life

**Table 4** presents the longitudinal changes in HRQoL among TACE-treated HCC survivors. Mauchly’s test indicated violations of sphericity for physical, psychological, social, and symptom domains (all *p* < 0.05), so Greenhouse–Geisser corrected degrees of freedom were used; the sphericity assumption was met for overall HRQoL (*W* = 0.990, *p* = 0.255). Social functioning showed no significant change over time (*p* = 0.588). Physical functioning (*p* = 0.011), psychological functioning (*p* < 0.001), symptom domain (*p* < 0.001), and overall HRQoL (*p* < 0.001) all changed significantly across the three time points. Symptom domain and overall HRQoL scores peaked at T2, declined moderately at T3, but remained higher than at T1 (T1 < T3 < T2). Psychological functioning improved continuously, reaching its highest level at T3 (T1 < T2 < T3). Physical functioning improved markedly at T2 and remained stable thereafter (T1 < T2).

**Table 4:** HRQoL domain scores across the three time points (*N* = 271).

Dimension	T1	T2	T3	*F*(df)	*p*	*Post-hoc* test
	(Mean ± SD)	(Mean ± SD)	(Mean ± SD)			
Physical function	4.27 ± 1.82	5.45 ± 1.91	5.18 ± 1.99	*F*(1.897, 512.207) = 4.645	0.011	T1 < T2
Psychological function	5.94 ± 1.65	6.61 ± 1.51	6.38 ± 1.63	*F*(1.953, 527.304) = 21.829	<0.001	T1 < T2 < T3
Social function	5.53 ± 1.16	5.61 ± 1.01	5.57 ± 1.08	*F*(1.789, 482.951) = 0.497	0.588	–
Symptom and side effect	7.18 ± 1.44	8.26 ± 0.88	8.06 ± 1.17	*F*(1.778, 480.022) = 90.770	<0.001	T1 < T3 < T2
Overall quality of life	4.56 ± 0.93	6.65 ± 1.04	5.24 ± 1.00	*F*(1.980, 534.600) = 559.209	<0.001	T1 < T3 < T2

### 3.6 Longitudinal variations in unmet needs

Longitudinal changes in unmet supportive care needs among HCC survivors after TACE are presented in **Table 5**. Mauchly’s test indicated violations of sphericity for information needs, work and financial needs, transitional care needs, and emotional and psychological support needs (all *p* < 0.05), so Greenhouse–Geisser corrected degrees of freedom were used; the sphericity assumption was met for total unmet needs (*W* = 0.990, *p* = 0.255). Significant time effects were observed for all subdomains and total scores (all *p* < 0.001). Unmet information needs and transitional care needs peaked at T3 (T1 < T3, T2 < T3). Unmet emotional and psychological support needs increased progressively across the three time points (T1 < T2 < T3), whereas unmet work and financial needs declined sequentially (T3 < T2 < T1). Total unmet needs reached their lowest level at T2, followed by a moderate rise at T1, and peaked at T3 (T2 < T1 < T3).

**Table 5:** Unmet needs domain scores across the three time points (*N* = 271).

Dimension	T1	T2	T3	*F*(df)	*p*	*Post-hoc* test
	(Mean ± SD)	(Mean ± SD)	(Mean ± SD)			
Information needs	1.96 ± 0.91	1.99 ± 0.81	2.36 ± 0.87	*F*(1.806, 487.552) = 43.934	<0.001	T1 < T3, T2 < T3
Work and financial needs	2.02 ± 0.58	1.39 ± 0.48	1.64 ± 0.57	*F*(1.795, 484.613) = 141.934	<0.001	T3< T2< T1
Transitional care needs	2.26 ± 0.64	2.26 ± 0.69	2.66 ± 0.71	*F*(1.617, 436.464) = 59.325	<0.001	T1 < T3, T2 < T3
Emotional and psychological support needs	0.96 ± 0.56	1.05 ± 0.53	1.23 ± 0.58	*F*(1.803, 486.688) = 36.472	<0.001	T1< T2< T3
Total unmet needs score	1.80 ± 0.46	1.67 ± 0.47	1.97 ± 0.49	*F (2*, 540) = 87.156	<0.001	T2<T1<T3

### 3.7 Correlation between quality of life and symptom clusters in HCC survivors after TACE

Spearman’s rank correlations between the five symptom clusters and HRQoL are presented in **Table 6**. All clusters showed negative correlations with overall HRQoL and its subdomains across the three time points, though not all were statistically significant (*r* = −0.904 to −0.085). The Somatic, Fatigue-Cognitive, and Emotional symptom clusters were significantly correlated with most HRQoL subdomains at every time point (all associations reached statistical significance). The magnitude of negative associations between the Somatic Symptom Cluster and overall HRQoL increased progressively from T1 to T3 (*r* = −0.583, −0.623, and −0.646, respectively). The Post-embolization Symptom Cluster, present only at T1, showed significant inverse correlations with all HRQoL domains (*r* = −0.271 to −0.477) and disappeared thereafter.

**Table 6:** Associations between symptom clusters and HRQoL (*N* = 271, *r*).

Dimension	Symptom Cluster	T1	T2	T3
Physical function	Somatic symptom cluster	−0.453**	−0.518**	−0.601**
	Fatigue-cognitive symptom cluster	−0.679**	−0.809**	−0.787**
	Emotional symptom cluster	−0.326**	−0.381**	−0.482**
	Hepatobiliary-specific symptom cluster	−0.085	−0.169**	−0.132*
	Post-embolization symptom cluster	−0.271**	–	–
Psychological function	Somatic symptom cluster	−0.452**	−0.449**	−0.473**
	Fatigue-cognitive symptom cluster	−0.326**	−0.395**	−0.375**
	Emotional symptom cluster	−0.848**	−0.904**	−0.830**
	Hepatobiliary-specific symptom cluster	−0.142*	−0.101	−0.169**
	Post-embolization symptom cluster	−0.356**	–	–
Social function	Somatic symptom cluster	−0.387**	−0.406**	−0.485**
	Fatigue-cognitive symptom cluster	−0.337**	−0.229**	−0.379**
	Emotional symptom cluster	−0.424**	−0.478**	−0.585**
	Hepatobiliary-specific symptom cluster	−0.232**	−0.175**	−0.301**
	Post-embolization symptom cluster	−0.463**	–	–
Symptom dimension	Somatic symptom cluster	−0.557**	−0.598**	−0.687**
	Fatigue-cognitive symptom cluster	−0.212**	−0.319**	−0.409**
	Emotional symptom cluster	−0.276**	−0.300**	−0.364**
	Hepatobiliary-specific symptom cluster	−0.134*	−0.268**	−0.253**
	Post-embolization symptom cluster	−0.477**	–	–
Overall quality of life	Somatic symptom cluster	−0.583**	−0.623**	−0.646**
	Fatigue-cognitive symptom cluster	−0.626**	−0.635**	−0.664**
	Emotional symptom cluster	−0.677**	−0.686**	−0.756**
	Hepatobiliary-specific symptom cluster	−0.195**	−0.208**	−0.229**
	Post-embolization symptom cluster	−0.459**	–	–

* p < 0.05, ** p < 0.01, indicating statistical significance of correlation coefficients.

### 3.8 Sensitivity analyses for confounding adjustment

To assess whether the primary longitudinal findings were influenced by potential confounders, we performed three linear mixed-effects models (LMMs) with participant-level random intercepts, adjusting for TNM stage, Child–Pugh class, and number of previous TACE sessions as fixed covariates.

First, for the HRQoL trajectory, the time effect remained highly significant after covariate adjustment [*F* (2, 804) = 317.47, *p* < 0.001]. Based on the estimated marginal means with T3 as the reference, HRQoL was significantly lower at T1 (*B* = −0.678, *p* < 0.001) and significantly higher at T2 (*B* = 1.417, *p* < 0.001), confirming the same temporal pattern as the primary RM-ANOVA: T1 < T3 < T2.

Second, for the unmet needs trajectory, the time effect also remained highly significant [*F* (2, 804) = 27.85, *p* < 0.001]. Using T3 as the reference, unmet needs were significantly lower at T1 (*B* = −0.173, *p* < 0.001) and at T2 (*B* = −0.301, *p* < 0.001), validating the same pattern as the primary analysis: T2 < T1 < T3.

Third, for the symptom–HRQoL association, the overall symptom severity score (averaged across the five EFA-derived symptom clusters) remained a significant predictor of HRQoL after adjusting for confounders [*F* (1, 801) = 1485.49, *p* < 0.001], confirming that greater symptom burden was independently associated with poorer HRQoL. A significant Time × Symptom interaction was also observed [*F* (2, 801) = 16.09, *p* < 0.001], indicating that the negative association was strongest at T2 and weakest at T1. These sensitivity results support the robustness of our primary conclusions.

## 4 Discussion

Through longitudinal follow-up, the present study tracked alterations in symptom clusters, core symptoms, and quality of life among HCC survivors after TACE across three recovery phases: acute (T1, 24 h post-procedure), subacute (T2, postoperative day 7), and early survivorship (T3, 1 month post-intervention). The overall symptom dynamics shifted from predominant physical symptom burden to increased psychological distress over the 1-month recovery period.

Five distinct symptom clusters consistent with the predefined classification were identified. The Post-embolization Symptom Cluster was observed exclusively at T1 and consisted of pain, nausea, vomiting, and fever as primary acute stress responses, which could spontaneously resolve alongside hepatic tissue repair and inflammatory resolution (23, 24). The Emotional Symptom Cluster, comprising distress and sadness, maintained a stable structure across all three time points. Previous studies have documented that HCC survivors endure significant psychological burden during treatment and recovery, and such negative affective states exacerbate physical discomfort, induce cognitive dysfunction, and hinder effective symptom management (25–27). Over half of our cohort had a history of hepatitis virus infection, which, combined with cumulative hepatic damage from repeated interventional treatments, may be associated with persistent bile metabolism disorders and long-term retention of the Hepatobiliary-Specific Symptom Cluster (jaundice and itching) (28). The Fatigue-Cognitive Symptom Cluster exhibited stable core characteristics across all time points, with fatigue, shortness of breath, and difficulty remembering persisting as dominant symptoms; by contrast, secondary symptoms, including dry mouth and numbness, declined as recovery progressed. This sustained burden aligns with prior TACE research by Zhang et al. (29). Somatic symptoms presented a progressive cumulative pattern: disturbed sleep, lack of appetite, and feeling bloated dominated the acute stage, while pain, nausea, and diarrhea were gradually incorporated at subacute and early survivorship stages. Our findings differ from those of Cao et al. (30), who found that disturbed sleep, lack of appetite, and feeling bloated existed as independent symptom dimensions without sustained clustering. This discrepancy likely reflects methodological differences between cross-sectional and longitudinal designs; Cao’s study compared symptom profiles only before and after TACE and failed to capture dynamic symptom restructuring during recovery. Furthermore, over half of our cohort had concurrent viral hepatitis—a chronic condition that impairs hepatic functional reserve and increases susceptibility to persistent physical symptoms. These observations suggest that unresolved acute symptoms may be linked to cascading reactions and amplify overall symptom burden.

Symptom network analysis validated regular sequential shifts in core symptoms and bridge nodes across the recovery continuum. At T1, nausea functioned as the core network node (rs = 1.154), mainly driven by the emetic effects of intra-arterial chemotherapeutic agents and acute perioperative psychological stress (31). As the dominant bridge node (rbs =3.767), lack of appetite mediated the transmission of gastrointestinal discomfort to multiple physical symptoms. This aligns with Xu et al. (32), who identified nausea and vomiting as central nodes in TACE recipients. At T2, lack of appetite replaced nausea as the core symptom (rs = 1.229). Resumed oral intake increased gastrointestinal load, while hepatic dysfunction from repeated TACE further aggravated digestive disturbances. Meanwhile, sadness emerged as the primary bridge node (rbs = 3.499), marking a transition from physical-dominant to psychological-dominant distress. This differs from Zhang et al. (33), who reported persistent physical symptom dominance before hospital discharge. This inconsistency can be explained by our cohort’s clinical characteristics: most survivors had advanced HCC (67.9% stage III–IV) and over 60% had received three or more TACE sessions. Repeated exposure to disease- and treatment-related stress may have accelerated the emergence of psychological symptoms. At T3, sadness peaked in both strength centrality and bridging influence (rs = 1.107, rbs = 4.140), becoming the central hub of the entire symptom network. Xu et al. (32) previously identified distress as the dominant central symptom; our study further confirms that sadness exerts a critical bridging effect in late-stage recovery. Nearly 40% of enrolled survivors had a disease duration exceeding 12 months. Protracted illness, uncertain prognosis, and cumulative persistent symptoms induced continuous psychological strain. As a key bridge node, sadness may further precipitate physical abnormalities, including disturbed sleep and diminished appetite, forming a bidirectional cycle linking physical and psychological symptoms.

This study also analyzed the longitudinal trajectories of HRQoL and unmet supportive care needs. Overall HRQoL scores exhibited a dynamic pattern: lowest at T1, significant improvement at T2, and a slight decline at T3 (T1 < T3 < T2, *p* < 0.001). No significant fluctuations were observed in physical and social functioning domains, indicating that HRQoL changes were primarily driven by symptom burden and psychological status. Consistent with our findings, Shun et al. (34). reported gradual post-discharge improvements in HRQoL among TACE-treated HCC survivors, with symptomatic distress and negative mood as key predictive factors. This temporal variation may have multiple explanations. At T1, severe post-embolization inflammatory reactions and acute psychological stress jointly suppressed HRQoL to its lowest level. At T2, the resolution of acute procedural symptoms and adaptive adjustments contributed to a marked elevation in HRQoL. At T3, persistent chronic hepatic injury and sadness-induced negative affect persisted; simultaneously, unmet supportive care needs continued to rise and reached their peak. These findings demonstrate that the transition from inpatient to home-based recovery is accompanied by insufficient symptom management, fragmented transitional care, and inadequate emotional support. This exposes survivors to overlapping physical, psychological, and unmet-care burdens, which may be associated with a mild decline in long-term HRQoL.

Based on our longitudinal evidence, we propose a staged, core symptom-targeted full-cycle survivorship management strategy for TACE-treated HCC survivors.

At T1 (acute stage), interventions should focus on mitigating nausea and vomiting while addressing the appetite-bridging effect of appetite loss. Early risk screening and integrated interventions may help to block potential cascading symptom aggravation and are worthy of consideration. Clinicians can combine antiemetic pharmacotherapy with non-pharmacological approaches, including music therapy, aromatherapy, and progressive muscle relaxation, alongside early dietary guidance and regulation of gastrointestinal function (35–37).

At T2 (subacute stage), interventions should prioritize alleviating appetite loss and preventing nutritional deficiencies. Routine psychological screening—using tools such as the Distress Thermometer (38, 39)—should be implemented to identify high-risk patients and potentially limit the bridging propagation of sadness. Individualized dietary regimens and nutritional supplementation should be provided based on clinical status, and timely psychological counseling should be delivered for individuals with abnormal mood with the aim of reducing the risk of reciprocal symptom aggravation.

At T3 (early survivorship stage), sadness acts as the absolute core of the symptom network, accompanied by peak unmet supportive care needs. This pattern suggests that psychological counseling and emotional support may warrant particular emphasis during this phase. Regular longitudinal psychological monitoring is recommended, with evidence-based approaches such as cognitive behavioral therapy and mindfulness meditation (40, 41). Hospital discharge planning should be optimized (42), and diversified measures—including standardized health education materials, remote online follow-up, and peer support groups (43, 44)—should be implemented to address gaps in information, guidance, emotional support, and transitional care. In addition, long-term monitoring of chronic symptoms in the Hepatobiliary-Specific, Fatigue-Cognitive, and Somatic symptom clusters should be maintained throughout survivorship.

Several limitations should be acknowledged. First, the single-center design may introduce selection bias, limiting generalizability. Second, the 1-month follow-up is relatively short, precluding observation of long-term symptom evolution. Third, temporal changes in symptom-network characteristics were only interpreted descriptively, without formal inferential statistical comparisons of overall network structure across time points. In addition, we did not collect ECOG performance-status data, which restricts our ability to fully characterize participants’ functional clinical status. Future multicenter, large-sample, long-term longitudinal studies are warranted to further refine individualized full-cycle rehabilitation strategies for TACE-treated HCC survivors.

## 5 Conclusion

This longitudinal network analysis identified distinct temporal symptom cluster trajectories and stage-specific core symptoms among patients with HCC after TACE. Nausea, appetite loss, and sadness were identified as key intervention targets across follow-up time points. Targeted, staged nursing interventions focusing on these core symptoms may help reduce cumulative symptom burden and optimize rehabilitation outcomes for HCC survivors receiving TACE.

## Data Availability

The raw data supporting the conclusions of this article will be made available by the authors, without undue reservation.
